# An Injectable Hydrogel for Enhanced FeGA-Based Chemodynamic Therapy by Increasing Intracellular Acidity

**DOI:** 10.3389/fonc.2021.750855

**Published:** 2021-09-22

**Authors:** Wen Zeng, Dazhen Jiang, Zeming Liu, Weilong Suo, Ziqi Wang, Daoming Zhu, Qinqin Huang

**Affiliations:** ^1^Department of Molecular Pathology, The Second Affiliated Hospital of Zhengzhou University, Zhengzhou, China; ^2^Department of Ophthalmology, Zhongnan Hospital of Wuhan University, Wuhan, China; ^3^Department of Radiation and Medical Oncology, Hubei Key Laboratory of Tumor Biological Behaviors, Hubei Cancer Clinical Study Center, Zhongnan Hospital of Wuhan University, Wuhan, China; ^4^Department of Plastic and Cosmetic Surgery, Tongji Hospital, Tongji Medical College, Huazhong University of Science and Technology, Wuhan, China; ^5^Key Laboratory of Artificial Micro- and Nano-Structures of Ministry of Education, School of Physics and Technology, Wuhan University, Wuhan, China

**Keywords:** FeGA, hydrogel, α-CHCA, chemodynamic therapy and tumor therapy, intracellular acidity

## Abstract

Hydroxyl radical (•OH)-mediated chemodynamic therapy (CDT) is an emerging antitumor strategy, however, acid deficiency in the tumor microenvironment (TME) hampers its efficacy. In this study, a new injectable hydrogel was developed as an acid-enhanced CDT system (AES) for improving tumor therapy. The AES contains iron–gallic acid nanoparticles (FeGA) and α-cyano-4-hydroxycinnamic acid (α-CHCA). FeGA converts near-infrared laser into heat, which results in agarose degradation and consequent α-CHCA release. Then, as a monocarboxylic acid transporter inhibitor, α-CHCA can raise the acidity in TME, thus contributing to an increase in ·OH-production in FeGA-based CDT. This approach was found effective for killing tumor cells both *in vitro* and *in vivo*, demonstrating good therapeutic efficacy. *In vivo* investigations also revealed that AES had outstanding biocompatibility and stability. This is the first study to improve FeGA-based CDT by increasing intracellular acidity. The AES system developed here opens new opportunities for effective tumor treatment.

## Introduction

Cancer, as one of the primary diseases affecting human health, has a profound impact on human life, and the patient’s condition becomes due to the rapid propagation and diffusion of cancer cells, and the lower efficacy of the currently used chemotherapeutic agents (1–5). However, cancer cells are very sensitive to reactive oxygen species (ROS), increased ROS levels can cause cell redox imbalance, thus resulting in permanent damage to the orgate and, eventually, apoptosis (6, 7). Based on this fact, researchers have developed a range of new treatment approaches to enhance tumor ROS levels, such as radiotherapy (RT), photodynamic therapy (PDT), CDT, and so on (8–10). However, the light-treated PDT is limited in the body, and long-term RT results in diverse side effects (11–13). Furthermore, increasing evidence suggests that the CDT for foreign iron treatment offers a significant advantage (14, 15). In TME, Fe^2+^ can react with enough hydrogen peroxide (H_2_O_2_), thus producing toxic •OH. Several other kinds of iron-containing formulations such as tetraned iron nanoparticles, iron oxide nanoparticles, and FeGA particles have been utilized as a catalyst for Fenton-reacted cell death, either alone or in combination with other catalysts (16, 17). Liu et al. used food acid and Fe^2+^ mixed ultra-small FeGA complex for achieving improved CDT (18). The catalytic stability of free Fe^2+^ was greatly improved due to GA-mediated Fe^3+^, and the combined glutathione (GSH) consumpant BSO was significantly improved as FeGA could consume GSH. This in turn improved oxidative stress in tumors, resulting in a considerable improvement in the treatment effect of simultaneous chemotherapy or radiotherapy. Although a significant anti-tumor efficacy is attained, the Fenton reaction’s effect is highly associated with the acidity of the tumor (19). With the decrease of pH, the reaction rate of Fe2+ with H_2_O_2_ increases. TME frequently exhibits a weak acid environment due to aberrant cancer cell metabolism and is unable to attain the optimal pH range of Fanton, which has a significant effect on CDT.

Cancer cells, unlike normal cells, are more likely to “ferment” glucose into lactic acid to produce adenosine triphosphate for energy rather than oxidative phosphorylation of mitochondria, even in normoxic conditions. This is the Warburg effect (20, 21). Furthermore, some tumor tissues continue to deteriorate lactic acid *via* the tricarboxylic acid (TCA) cycle to prevent long-term accumulation of lactic acid in the cells, thus inducing cell disorders. This is the metabolic process that tumor cells go through (22). As a result, disrupting the tumor’s metabolic equilibrium by affecting the cyclic impact of TCG or the lactic acid transfer chain will eventually result in a substantial lactic acid accumulation in the cell and leads to cell acidosis (23). This in turn results in improved anti-tumor effects. Wan et al. used α-CHCA, a monocarboxylic acid transport inhibitor, to destroy the delivery process of lactic acid, alter the metabolic homeostasis, and used it in combination with H_2_S to achieve a powerful tumor-killing effect (23). This approach only targets cancer tissues during therapy and does not produce inflammatory reactions or other organ side effects, thus demonstrating a high level of biological safety. However, most small-molecule drugs are administered *via* intravenous injection. The drugs are incapable of evading the immune system and cannot actively target tumor cells (24). A considerable number of drugs are removed through the bloodstream, and the drugs reaching the tumor are uncontrolled. These factors greatly limit the treatment efficacy.

Traditional drug delivery systems usually have issues such as poor drug loading, difficult manufacturing processes, and early drug leakage or slow release (25–27). Although many inorganic and organic materials are also widely used in biological applications, most of them are injected intravenously, with high material loss rate and difficulty in reaching tumor tissues (28). Moreover, it is difficult to achieve controlled release of these materials or require complex modifications to improve their biological applicability, which is not conducive to clinical applications (29, 30). The long-term toxicity induced by the carrier persisting in the body for an extended period is also an important aspect. Light-responsive hydrogels with minimal invasiveness have recently gained popularity as a controlled drug release platform (31–33). The hydrogel gradually solidifies after being injected into tumor tissue and can be utilized as a military rationing depot for a longer period, this characteristic is attributed to the rapid decrease of the temperature of the hydrogel and the increase of the storage modulus, which leads to the solidification of the hydrogel and its long-term residence in the body (34, 35). After one injection, this form of local administration can be used again and again. Furthermore, the drug release rate can be changed by optimizing parameters such as laser strength and laser irradiation period and extending the treatment method’s application. For the first time, Zhu et al. used an agarose hydrogel to deliver the AIEgen material for anti-tumor treatment. As a photothermal agent (PTA), prussian blue (PB) nanozyme stimulated the disintegration of the hydrogel while also acting as a CAT enzyme to catalyze H_2_O_2_ for improving the TME (31). Following that, a low-power white light was used. AIEgens produced ROSs under sufficient oxygen levels for promoting tumor ablation upon irradiation. As a result of these findings, we are encouraged to use hydrogels to deliver α-CHCA for disrupting the tumor’s ecological balance and boosting the efficacy of FeGA-based CDT.

**Scheme 1:**
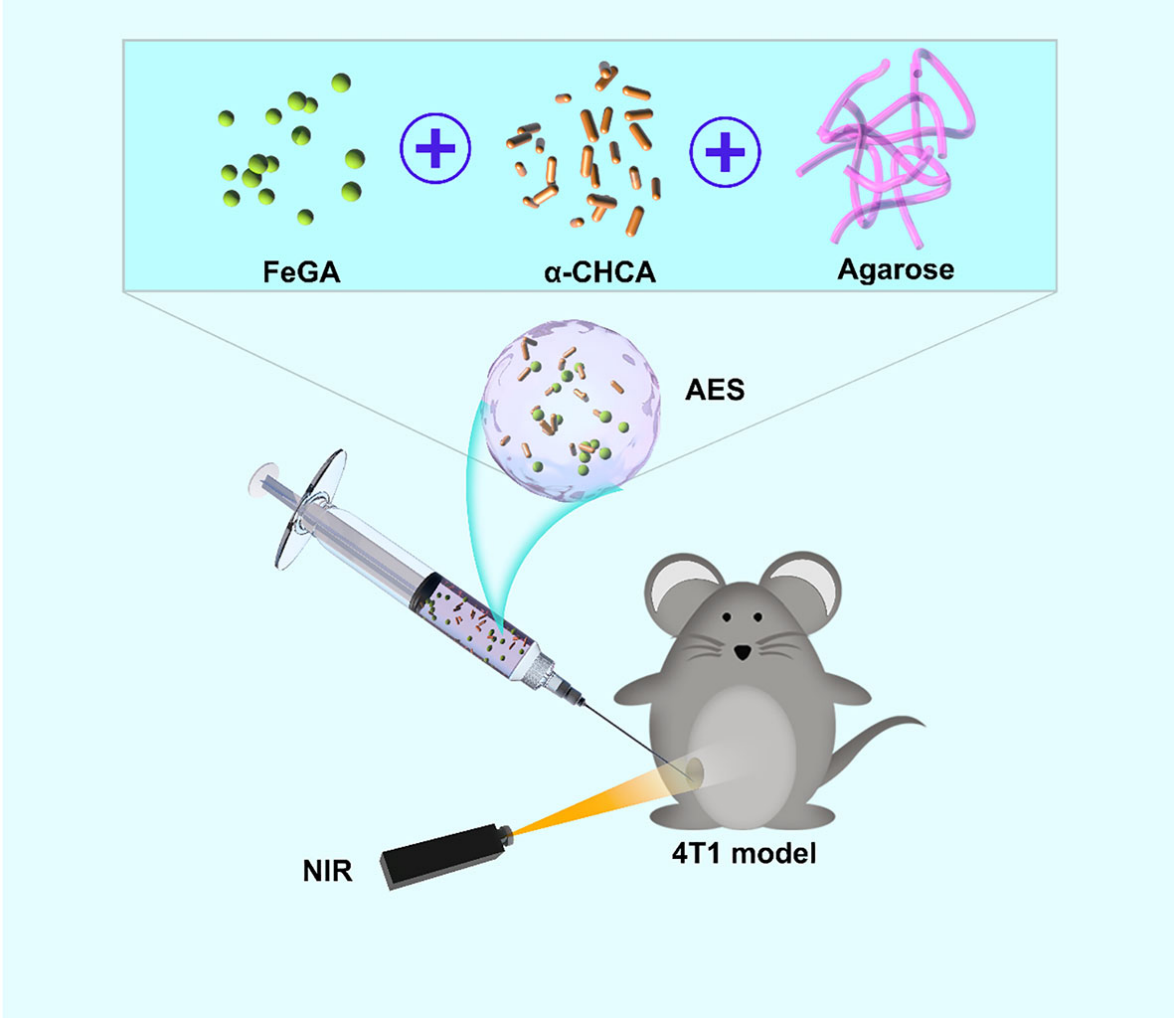
Schematic illustration of an injectable hydrogel for enhanced FeGA-based chemodynamic therapy by increasing intracellular acidity.

We designed an injectable hydrogel with FeGA nanoparticles and α-CHCA for intratumoral injection of chemodynamic and photothermal therapy (**Scheme 1**). The US Food and Drug Administration (FDA) has declared agarose hydrogels to be safe. FeGA nanoparticles and α-CHCA were placed into an agarose hydrogel for producing the FeGA reservoir and acid enhance system (AES). In this system, the FeGA nanoparticles serve as a PTA due to their outstanding photothermal performance. FeGA turns light energy into heat energy upon irradiating the AES system with an 808 nm near-infrared (NIR) laser, thus causing the temperature rise of the agarose hydrogel, as a result, reversible hydrolysis and softening occur. α-CHCA diffuses into the TME, inhibiting lactic acid efflux and intracellular accumulation in tumor cells, resulting in tumor acidosis. The Fenton reaction, which is driven by Fe^2+^, can then produce a high amount of •OH, which can damage tumor cells. The AES can be employed as a FeGA storage controller for achieving the controlled release of the drug and for intratumor injection of local tumors. This is the first study to show that increasing intracellular acidity *in situ* improves FeGA-based CDT. In conclusion, the FH nanosystem has a wide range of clinical applications in synergetic therapy.

## Results and Discussion

### Characterization of FeGA Nanoparticles and AES

**Figure 1:**
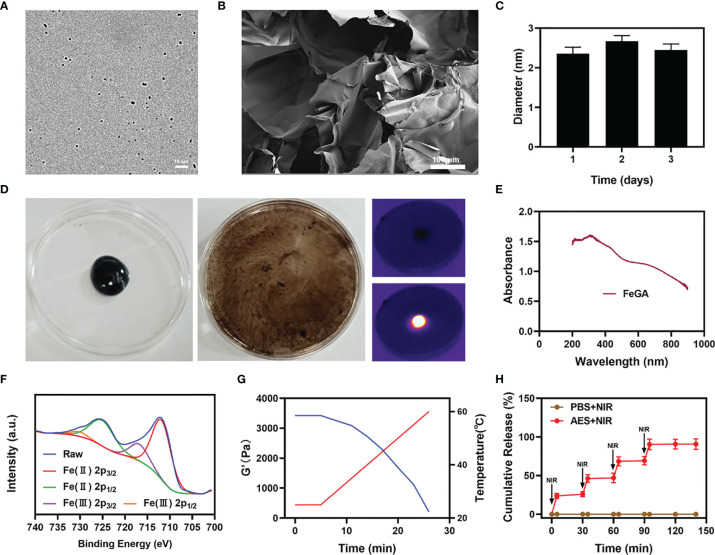
Characterization of AES. **(A)** TEM image of FeGA. **(B)** SEM image of the hydrogel. **(C)** Statistical graph of the measured diameter of FeGA. (n = 3). **(D)** The morphology of the prepared AES before and after 0.5 W/cm^2^ 808 nm laser irradiation for 10 min and infrared thermal images of the prepared AES after being irradiated. **(E)** FeGA absorbance spectra. **(F)** Fe 2p spectrum of XPS spectra of fresh GA–Fe. **(G)** Rheological and temperature curves (blue and red, respectively) for the prepared AES in response to 0.5 W/cm^2^ 808 nm laser irradiation. **(H)** *In vitro* AES release profile in the presence and absence of 808 nm laser irradiation, with red arrows being used to indicate irradiation time points (n = 3). The results were expressed as mean ± SD.

**Figure 1A** shows a transmission electron microscope image of FeGA. The results show better dispersibility and smaller size of FeGA, with an average of 2.36 ± 1.2 nm. The nanoparticles of smaller than 10 nm in size are easily cleared by the kidney, which limits its therapeutic efficacy. Therefore, the applicability of FeGA can be greatly improved through the hydrogel delivery system. The particle size of FeGA was measured for three consecutive days (**Figure 1C**), and the size fluctuation range was found small, reflecting the good stability of FeGA. Thus, in turn makes it a good system in clinical application prospects. Even though many materials have a beneficial biological impact, their high instability restricts their future value (36). The agarose hydrogel has been authorized by the FDA as a safe material that does not cause any toxicity in the body and is metabolized by the body’s natural processes following dissolution. The scanning electron microscope (SEM) image of the agarose hydrogel is shown in **Figure 1B**. The pore size of the hydrogel is larger and the AES system was then developed by encapsulating FeGA and α-CHCA in a hydrogel. **Figure 1D** demonstrates the good photothermal heating potential of AES while the developed hydrogel remains solid. The hydrogel gradually dissolves after 10 minutes of 808 nm laser irradiation. The drug and nanoparticles in the gel are almost totally dissolved and are released. Infrared thermal imaging further indicated that following irradiation, the temperature of AES increased dramatically. **Figure 1E** shows the ultraviolet-visible absorption of FeGA. FeGA has a quite high absorption bandwidth in the 600-800 nm range, with a distinctive peak near 600 nm. X-ray photoelectron spectroscopy (XPS) was used to measure the Fe 2p spectrum in FeGA (**Figure 1F**). Zeta potential of FeGA was detected to be -18.1 ± 6.57mV. The rheological value of AES was evaluated at various temperatures (**Figure 1G**), and the results revealed that as the temperature increases, AES rapidly dissolves, accompanied by a gradual decrease in storage modulus. This is in line with the hydrogel’s rheological properties. We then put AES to the test to see if it could regulate the release of materials and the results are described in **Figure 1H**. Laser irradiation can partially disintegrate AES and liberate the α-CHCA within it. The hydrogel becomes cool and hardens after the laser irradiation is stopped, and the drug will remain protected. The drug is usually released completely after four laser switching cycles. This also demonstrates that our AES system has a strong ability to control drug release, which inhibits lactic acid efflux, strengthens the tumor’s acidic environment, and keeps the cells in an acidic environment, which is likely to promote FeGA-mediated CDT.

### Photo-Thermal of the FeGA for PTT

**Figure 2:**
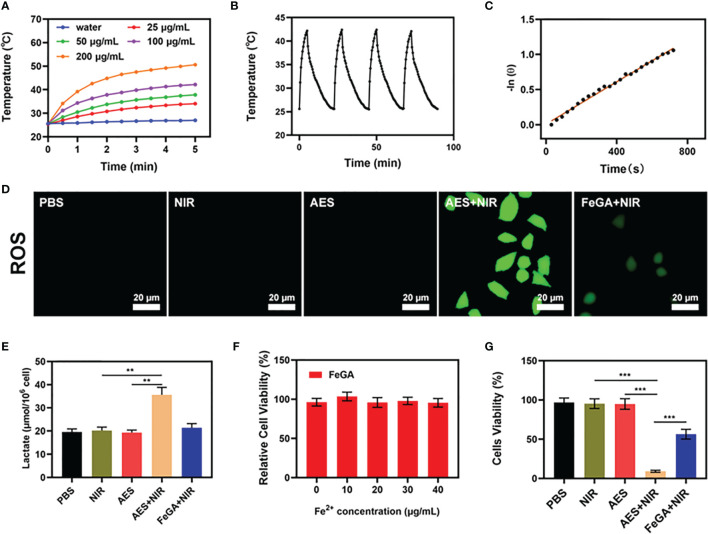
*In vitro* synergetic therapeutic effects of the AES. **(A)** Temperature increase for the different FeGA concentrations upon laser irradiation at 808 nm and 0.5 W/cm^2^ for 5min. **(B)** Temperature variation of a FeGA solution at 100 μg/mL under cyclic laser irradiation. **(C)** Calculation of the time constant for the heat transfer using linear regression of the cooling profile. **(D)** Measurement of tumor cell ROS fluorescence after the indicated treatments (n = 5). **(E)** *In vitro* lactate accumulation effect of different formulations (n = 5). **(F)** Dark cytotoxicity of FeGA on 4T1 cells (n = 5). **(G)** *In vitro* cytotoxicity of different formulations against 4T1 cells (n = 5). **P < 0.01, ***P < 0.005; Student’s t-test. The results were expressed as mean ± SD.

One of the most essential factors for evaluating PTA is photothermal stability. A powerful photothermal treatment can be assisted by a good photothermal agent. To test the photothermal performance of FeGA nanoparticles, FeGA solutions were prepared with different concentrations (0, 25, 50, 100, 200 μg/mL). **Figure 2A** shows that assuming all other parameters remain constant, the heating impact of the solution increases as the FeGA concentration rises. The temperature of 100 μg/mL FeGa increased by roughly 16.6°C after 5 min of laser irradiation. For 5 minutes, the 200 μg/mL FeGA solution was repeatedly heated with the 808 nm NIR laser (**Figures 2B, D, E**), then the solution was allowed to cool to ambient temperature. The heating curves of each cycle were identical, and the variations in peak temperature changes were minor, demonstrating that the FeGA nanoparticles’ photothermal conversion capability was found to be stable and reproducible over repeated 4 heating and cooling cycles. These findings suggest that the FeGA nanoparticles have good photothermal stability. Furthermore, the photo-thermal conversion efficiency (η) of the FeGA was calculated from the data of **Figures 2C, F**, and was found as 42.6%, which was higher than various materials such as Au nanorods (21%) and Ti_3_C_2_ nanosheets (30.6%) (37, 38). Above all, FeGA nanoparticles are an excellent PTA for photothermal conversion to anti-tumor.

### *In Vitro* Combination Therapy

Furthermore, we assessed the ROS content of several formulations. There is no green fluorescence in the control group, the NIR group, or the AES group when treated alone. The AES + NIR group had high fluorescence, whereas the FeGA + NIR group had much lower fluorescence. This could be because α-CHCA alters the tumor acid microenvironment, and FeGA reacts with enough H_2_O_2_ to produce increased ROS. ROS can cause cellular protein and DNA damage, resulting in the death of tumor cells (39). In several experimental groups, we continued to evaluate the lactic acid concentration of the TME. The results revealed that, despite the presence of α-CHCA in the AES group, α-CHCA was unable to affect the cells due to the hydrogel’s encapsulation, but the AES + NIR group had increased lactic acid concentration. α-CHCA can be released after laser irradiation, changing the lactic acid ecological microenvironment of tumor cells. Hence, the AES + NIR group was able to produce good lactate accumulating effect. The acidic environment is conducive to the subsequent FeGA-mediated Fenton reaction. FeGA was incubated with 4T1 cells at various concentrations (0, 10, 20, 30, 40 μg/mL) for 24 hours. Even at high concentrations, cell viability did not decrease significantly. The findings suggested that FeGA NPs are highly biocompatible. The cell viability of the control group was largely unaffected by the MTT experiment, whereas the NIR with FeGA group demonstrated a little inhibition in tumor growth (**Figure 2G**). AES + NIR system exhibited the best tumor growth inhibition rate (about 88.5%), with significant differences compared to other experiment groups indicating that AES mediated controlled release of α-CHCA can effectively increase the acidity of TME, thereby enhancing the CDT effect and inhibiting tumor growth. These findings motivate our ongoing research to proceed with the developed formulation for anti-tumor efficacy *in vivo*.

### *In Vivo* Anti-tumor Study

**Figure 3:**
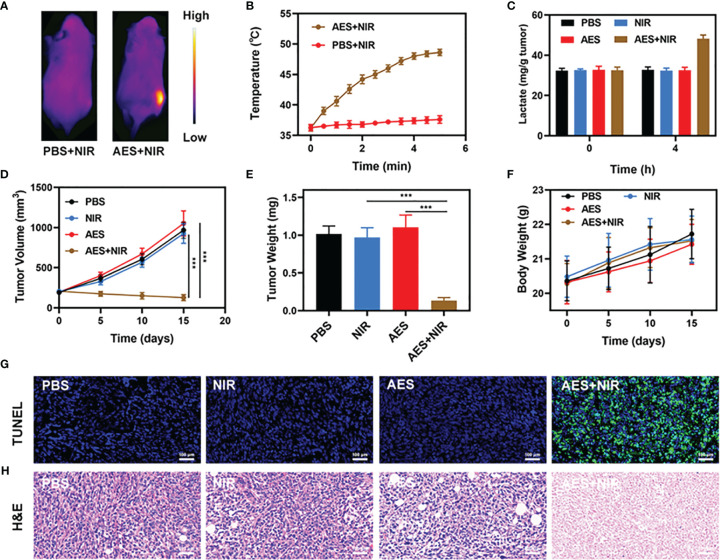
*In vivo* therapy. **(A)** IR thermal images of tumors following an 808 nm laser irradiation (0.5 W/cm^2^) for 5 min in the indicated treatment groups (n = 3). **(B)** Temperature increase in mice implanted with 4T1 tumors following 808 nm laser irradiation (0.5 W/cm^2^) for 5 min in the indicated treatment groups. **(C)** Lactate accumulation effect of different *in vivo* treatments (n = 5). **(D)** Tumor volume changes over time in groups treated as indicated (n = 5). **(E)** Average tumor weight values associated with the indicated treatments (n = 5). **(F)** Changes in body weight in response to the indicated treatments (n = 5). **(G)** TUNEL and **(H)** H&E stained tumor sections from the indicated treatment groups (n = 5). ***P < 0.005; Student’s t-test. The results were expressed as mean ± SD.

As stated earlier, the good performance of AES *in vitro* as a PTA and acid boosting system has prompted us to investigate the photothermal conversion effect of FeGA *in vivo*. BALB/c mice were used to establish 4T1 subcutaneous tumor models. After invading the tumor tissue, we measured the temperature rise of AES under laser irradiation. The PBS group rarely heated up after laser irradiation, as seen in **Figures 3A, B**, whereas the temperature of the tumor in the AES group increased dramatically, showing that AES has strong photothermal performance. This finding is similar to the *in vitro* photothermal findings, demonstrating that AES can provide *in vivo* photothermal therapy and drug release. Tumor tissues’ heat resistance was lowered when compared to normal cells, resulting in tumor cell-selective apoptosis at high temperatures (42 – 47°C) (17). Following that, we measured the amount of lactic acid in the tumor after various treatments. The lactic acid level of the tumor increased considerably after 4 hours of AES plus NIR treatment, as seen in **Figure 3C**. However, no obvious signals of increase were observed in the other groups, demonstrating that the released -CHCA can effectively prevent tumor cell lactic acid efflux and intracellular accumulation, leading to tumor acidosis (**Figure 3C**). The anti-tumor efficacy of AES-mediated anti-tumor therapy was next evaluated in mice with 4T1 tumors. BALB/c mice were injected subcutaneously with 1 × 10^6^ 4T1 cells in the right flank to assess the main effect of AES. The mice were treated after reaching the primary tumor volume to 200 mm^3^. Tumor-bearing mice were randomly divided into 4 groups (5 mice per group): (1) PBS; (2) NIR; (3) AES and (4) AES + NIR. The equivalent FeGA dose was 5 mg/kg in groups 3 and 4. For 16 days, treatment was given every 4 days. The body mass of treated and control mice remained normal after treatment, showing the safety of our technique (**Figure 3F**). This is quite interesting because many treatments are associated with severe systemic toxicity, which is detrimental to the material’s future clinical application (40). The tumor volumes of the PBS and NIR treated groups increased substantially during the 2 weeks of treatment, as illustrated in **Figure 3D**. In addition, the AES group had essentially no tumor-suppressive effect. The hydrogel entrapped FeGA and CHCA are unable to kill tumors. The AES + NIR system, which included both FeGA, had the most potent therapeutic response as the tumor volume growth curves being nearly reduced during therapy (**Figures 3D, E**). When the AES hydrogel is exposed to laser radiation after intratumoral injection, it dissolves and releases FeGA and α-CHCA in FH. α-CHCA would raise the tumor’s lactic acid concentration. FeGA then combines with intratumoral H_2_O_2_ to produce •OH in situ, which kills the tumor. We tested the fluorescence intensity of ROS in different groups *in vivo*, and the results also showed that AES + NIR group showed the strongest green fluorescence, and produced a large amount of ROS to promote tumor apoptosis. The tumor mass of mice was also in agreement with the volume curve (**Figure 3E**). We obtained slices of tumor tissue for staining. TUNEL and H&E staining (**Figures 3G, H**) also confirmed the large amount of cell necrosis in the AES combined NIR treatment group.

### Histological Analysis

**Figure 4:**
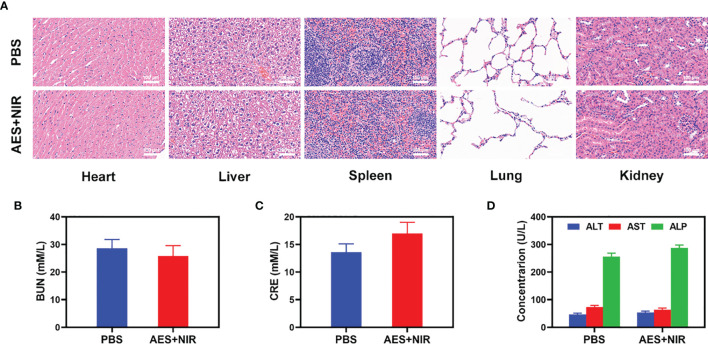
Result of *in vivo* safety experiments. **(A)** Histopathological analysis results (H&E-stained images) of the major organs, heart, lung, liver, kidneys, and spleen, of mice that were exposed to different treatments of 16 days post-injection. Blood biochemistry data including kidney function markers: **(B)** liver function markers: BUN, **(C)** CRE, and **(D)** ALT, ALP, and AST after various treatments (n = 5). The results were expressed as mean ± SD.

Furthermore, FeGA activation did not cause any loss to systems, as shown in **Figure 4**. After the treatment of mice’s vital organs (heart, liver, spleen, lungs, and kidney), there was no inflammation, and damage in the body, and the liver, and kidney indexes were also normal. As many nanomaterials possess great therapeutic efficacy, they are also associated with systemic toxicity, which limits their future clinical applications (41). *In vivo* data show that our novel combination therapy not only achieves a high level of biological safety but also increases tumor •OH content, thus enhancing the effect of FH-enhanced therapy.

## Conclusion

Finally, we developed an injectable light-controlled hydrogel system, called an acid to enhance system AES, by encapsulating FeGA nanoparticles and α CHCA in agarose hydrogel. The nano-system can be combined with 808 nm laser irradiation for achieving outstanding tumor treatment effects. FeGA nanoparticles can be employed as an ideal PTA due to their superior photothermal effect in the NIR-I region. The agarose hydrogel underwent controlled and reversible hydrolysis and softening states under the NIR laser power, resulting in light-triggered FeGA nanoparticles release and degradation of hydrogel. Then, α-CHCA would then cause tumor acidosis in the region, causing an increase in the Fenton reaction. The AES exhibits outstanding cancer cell killing and tumor ablation properties in both *in vitro* and *in vivo* tests, with good stability, low toxicity, and biocompatibility. This is the first study to show that increasing intracellular acidity in FeGA-based CDT improves its performance. Thus, we may conclude that the AES has great anti-cancer potential in combination therapy.

## Data Availability Statement

The raw data supporting the conclusions of this article will be made available by the authors, without undue reservation.

## Ethics Statement

The animal experiments were carried out according to the protocol approved by the Ministry of Health in People’s Republic of PR China and were approved by the Administrative Committee on Animal Research of the Zhengzhou University.

## Author Contributions

Conceived and designed the experiments: ZW, WZ, ZL, WS, DJ, and QH. Performed the experiments: WZ, DZ, ZL, ZW, and WS. Contributed reagents/materials/analysis tools: ZW, WS, WZ, DJ and QH. All authors contributed to the article and approved the submitted version.

## Funding

This work was supported by the National Natural Science Foundation of China (31800085) and Zhongnan Hospital of Wuhan University Science, Technology and Innovation Seed Fund, Project znpy2019022.

## Conflict of Interest

The authors declare that the research was conducted in the absence of any commercial or financial relationships that could be construed as a potential conflict of interest.

## Publisher’s Note

All claims expressed in this article are solely those of the authors and do not necessarily represent those of their affiliated organizations, or those of the publisher, the editors and the reviewers. Any product that may be evaluated in this article, or claim that may be made by its manufacturer, is not guaranteed or endorsed by the publisher.

## Supplementary Material

Supplementary Figures S1-4 Representative immunofluorescence images of ROS in tumor slices.

## References

[1] Zhu D Lyu M Jiang W Suo M Huang Q Li K. A Biomimetic Nanozyme/Camptothecin Hybrid System for Synergistically Enhanced Radiotherapy. J Materr Chem B (2020) 8:5312–9. doi: 10.1039/D0TB00676A 32453333

[2] Zhu D Lyu M Huang Q Suo M Liu Y Jiang W. Stellate Plasmonic Exosomes for Penetrative Targeting Tumor NIR-II Thermo-Radiotherapy. ACS Appl Materr Interfaces (2020) 12(33):36928–37. doi: 10.1021/acsami.0c09969 32814380

[3] Huang C Wang FB Liu L Jiang W Liu W Ma W. Hypoxic Tumor Radiosensitization Using Engineered Probiotics. Adv Healthcare Materr (2021) 10:2002207. doi: 10.1002/adhm.202002207 33645010

[4] Ding S Liu Z Huang C Zeng N Jiang W Li Q. Novel Engineered Bacterium/Black Phosphorus Quantum Dot Hybrid System for Hypoxic Tumor Targeting and Efficient Photodynamic Therapy. ACS Appl Materr Interfaces (2021) 13(8):10564–73. doi: 10.1021/acsami.0c20254 33605723

[5] Yu W Liu T Zhang M Wang Z Ye J Li CX. O2 Economizer for Inhibiting Cell Respiration To Combat the Hypoxia Obstacle in Tumor Treatments. ACS Nano (2019) 13(2):1784–94. doi: 10.1021/acsnano.8b07852 30698953

[6] Cheng Y-J Qin S-Y Ma Y-H Chen X-S Zhang A-Q Zhang X-Z. Super-pH-Sensitive Mesoporous Silica Nanoparticle-Based Drug Delivery System for Effective Combination Cancer Therapy. ACS Biomater Sci Eng (2019) 5(4):1878–86. doi: 10.1021/acsbiomaterials.9b00099 33405561

[7] Liu LH Zhang YH Qiu WX Zhang L Gao F Li B. Dual-Stage Light Amplified Photodynamic Therapy Against Hypoxic Tumor Based on an O2 Self-Sufficient Nanoplatform. Small (2017) 13(37):1701621. doi: 10.1002/smll.201701621 28783253

[8] Guo J Huang L. Membrane-Core Nanoparticles for Cancer Nanomedicine. Adv Drug Delivery Rev (2020) 156:23–39. doi: 10.1016/j.addr.2020.05.005 PMC7680273 32450105

[9] Wang X-Q Wang W Peng M Zhang X-Z. Free Radicals for Cancer Theranostics. Biomaterials (2021) 266:120474. doi: 10.1016/j.biomaterials.2020.120474 33125969

[10] Li S Tan L Meng X. Nanoscale Metal-Organic Frameworks: Synthesis, Biocompatibility, Imaging Applications, and Thermal and Dynamic Therapy of Tumors. Adv Funct Mater (2020) 30(13):1908924. doi: 10.1002/adfm.201908924

[11] Zhao LP Zheng RR Chen HQ Liu LS Zhao XY Liu HH. Self-Delivery Nanomedicine for O2-Economized Photodynamic Tumor Therapy. Nano Lett (2020) 20(3):2062–71. doi: 10.1021/acs.nanolett.0c00047 32096643

[12] Ren SZ Wang B Zhu XH Zhu D Liu M Li SK. Oxygen Self-Sufficient Core-Shell Metal-Organic Framework-Based Smart Nanoplatform for Enhanced Synergistic Chemotherapy and Photodynamic Therapy. ACS Appl Materr Interfaces (2020) 12(22):24662–74. doi: 10.1021/acsami.0c08534 32394704

[13] Jin C Bai L Wu H Tian F Guo G. Radiosensitization of Paclitaxel, Etanidazole and Paclitaxel+Etanidazole Nanoparticles on Hypoxic Human Tumor Cells In Vitro. Biomaterials (2007) 28(25):3724–30. doi: 10.1016/j.biomaterials.2007.04.032 17509678

[14] Fu S Yang R Zhang L Liu W Du G Cao Y. Biomimetic CoO@AuPt Nanozyme Responsive to Multiple Tumor Microenvironmental Clues for Augmenting Chemodynamic Therapy. Biomaterials (2020) 257:120279. doi: 10.1016/j.biomaterials.2020.120279 32763613

[15] Meng X Jia K Sun K Zhang L Wang Z. Smart Responsive Nanoplatform via in Situ Forming Disulfiram-Copper Ion Chelation Complex for Cancer Combination Chemotherapy. Chem Eng J (2021) 415:128947. doi: 10.1016/j.cej.2021.128947

[16] Zhang X He C Chen Y Chen C Yan R Fan T. Cyclic Reactions-Mediated Self-Supply of H2O2 and O2 for Cooperative Chemodynamic/Starvation Cancer Therapy. Biomaterials (2021) 275:120987. doi: 10.1016/j.biomaterials.2021.120987 34175561

[17] He Y Guo S Zhang Y Liu Y Ju H. NIR-II Reinforced Intracellular Cyclic Reaction to Enhance Chemodynamic Therapy With Abundant H2O2 Supply. Biomaterials (2021) 275:120962. doi: 10.1016/j.biomaterials.2021.120962 34153782

[18] Dong Z Feng L Chao Y Hao Y Chen M Gong F. Amplification of Tumor Oxidative Stresses With Liposomal Fenton Catalyst and Glutathione Inhibitor for Enhanced Cancer Chemotherapy and Radiotherapy. Nano Lett (2019) 19(2):805–15. doi: 10.1021/acs.nanolett.8b03905 30592897

[19] Li M Lan X Han X Shi S Sun H Kang Y. Acid-Induced Self-Catalyzing Platform Based on Dextran-Coated Copper Peroxide Nanoaggregates for Biofilm Treatment. ACS Appl Materr Interfaces (2021) 13(25):29269–80. doi: 10.1021/acsami.1c03409 34143595

[20] Liberti MV Locasale JW. The Warburg Effect: How Does it Benefit Cancer Cells? Trends Biochem Sci (2016) 41(3):211–8. doi: 10.1016/j.tibs.2015.12.001 PMC4783224 26778478

[21] Icard P Shulman S Farhat D Steyaert JM Alifano M Lincet H. How the Warburg Effect Supports Aggressiveness and Drug Resistance of Cancer Cells? Drug Resist Updates Rev Commentaries Antimicrob Anticancer Chemother (2018) 38:1–11. doi: 10.1016/j.drup.2018.03.001 29857814

[22] Gao F Tang Y Liu WL Zou MZ Huang C Liu CJ. Intra/Extracellular Lactic Acid Exhaustion for Synergistic Metabolic Therapy and Immunotherapy of Tumors. Adv Materr (2019) e1904639. doi: 10.1002/adma.201904639 31692128

[23] Chen Q-W Wang J-W Wang X-N Fan J-X Liu X-H Li B. Inhibition of Tumor Progression Through the Coupling of Bacterial Respiration With Tumor Metabolism. Angewandte Chemie (International Ed English) (2020) 59(48):21562–70. doi: 10.1002/anie.202002649 32779303

[24] Huang C Ding S Jiang W Wang FB. Glutathione-Depleting Nanoplatelets for Enhanced Sonodynamic Cancer Therapy. Nanoscale (2021) 13:4512–8. doi: 10.1039/D0NR08440A 33615325

[25] Ren C Cheng Y Li W Liu P Yang L Lu Q. Ultra-Small Bi2S3 Nanodot-Doped Reversible Fe(ii/iii)-Based Hollow Mesoporous Prussian Blue Nanocubes for Amplified Tumor Oxidative Stress-Augmented Photo-/Radiotherapy. Biomater Sci (2020) 8(7):1981–95. doi: 10.1039/C9BM02014D 32068203

[26] Jin L Hu P Wang Y Wu L Qin K Cheng H. Fast-Acting Black-Phosphorus-Assisted Depression Therapy With Low Toxicity. Adv Materr (2020) 32(2):e1906050. doi: 10.1002/adma.201906050 31777995

[27] Zhu Y Shi H Li T Yu J Guo Z Cheng J. Functional Nanoreactor for Synergistic Starvation and Photodynamic Therapy. ACS Appl Materr Interfaces (2020) 12(16):18309–18. doi: 10.1021/acsami.0c01039 32233414

[28] Wang J Sui L Huang J Miao L Nie Y Wang K. MoS2-Based Nanocomposites for Cancer Diagnosis and Therapy. Bioact Mater (2021) 6(11):4209–42. doi: 10.1016/j.bioactmat.2021.04.021 PMC8102209 33997503

[29] Huang Q Wu W Ai K Liu J. Highly Sensitive Polydiacetylene Ensembles for Biosensing and Bioimaging. Front Chem (2020) 8:565782. doi: 10.3389/fchem.2020.565782 33282824 PMC7691385

[30] Wang Y Pei H Jia Y Liu J Li Z Ai K. Synergistic Tailoring of Electrostatic and Hydrophobic Interactions for Rapid and Specific Recognition of Lysophosphatidic Acid, an Early-Stage Ovarian Cancer Biomarker. J Am Chem Soc (2017) 139(33):11616–21. doi: 10.1021/jacs.7b06885 28782946

[31] Zhu D Zheng Z Luo G Suo M Li X Duo Y. Single Injection and Multiple Treatments: An Injectable Nanozyme Hydrogel as AIEgen Reservoir and Release Controller for Efficient Tumor Therapy. Nano Today (2021) 37:101091. doi: 10.1016/j.nantod.2021.101091

[32] Huang C Chen B Chen M Jiang W Liu W. Injectable Hydrogel for Cu(2+) Controlled Release and Potent Tumor Therapy. Life (Basel) (2021) 11(5):391. doi: 10.3390/life11050391 33925834 PMC8147102

[33] Chen D Chen C Huang C Chen T Liu Z. Injectable Hydrogel for NIR-II Photo-Thermal Tumor Therapy and Dihydroartemisinin-Mediated Chemodynamic Therapy. Front Chem (2020) 8:251. doi: 10.3389/fchem.2020.00251 32318547 PMC7154176

[34] Meng Z Zhou X Xu J Han X Dong Z Wang H. Light-Triggered In Situ Gelation to Enable Robust Photodynamic-Immunotherapy by Repeated Stimulations. Adv Materr (2019) 31(24):e1900927. doi: 10.1002/adma.201900927 31012164

[35] Qiu M Wang D Liang W Liu L Zhang Y Chen X. Novel Concept of the Smart NIR-Light-Controlled Drug Release of Black Phosphorus Nanostructure for Cancer Therapy. Proc Natl Acad Sci USA (2018) 115(3):501–6. doi: 10.1073/pnas.1714421115 PMC5776980 29295927

[36] Chang M Hou Z Jin D Zhou J Wang M Wang M. Colorectal Tumor Microenvironment-Activated Bio-Decomposable and Metabolizable Cu2 O@CaCO3 Nanocomposites for Synergistic Oncotherapy. Adv Materr (2020) 32(43):2004647. doi: 10.1002/adma.202004647 32945002

[37] Song G Liang C Gong H Li M Zheng X Cheng L. Core-Shell MnSe@Bi2 Se3 Fabricated via a Cation Exchange Method as Novel Nanotheranostics for Multimodal Imaging and Synergistic Thermoradiotherapy. Adv Materr (2015) 27(40):6110–7. doi: 10.1002/adma.201503006 26331476

[38] Chen G Yang Y Xu Q Ling M Lin H Ma W. Self-Amplification of Tumor Oxidative Stress With Degradable Metallic Complexes for Synergistic Cascade Tumor Therapy. Nano Lett (2020) 20(11):8141–50. doi: 10.1021/acs.nanolett.0c03127 33172280

[39] Zhang X Li G Wu D Li X Hu N Chen J. Recent Progress in the Design Fabrication of Metal-Organic Frameworks-Based Nanozymes and Their Applications to Sensing and Cancer Therapy. Biosensors Bioelectron (2019) 137:178–98. doi: 10.1016/j.bios.2019.04.061 31100598

[40] Zhou J Wang M Han Y Lai J Chen J. Multistage-Targeted Gold/Mesoporous Silica Nanocomposite Hydrogel as In Situ Injectable Drug Release System for Chemophotothermal Synergistic Cancer Therapy. ACS Appl Bio Mater (2019) 3(1):421–31. doi: 10.1021/acsabm.9b00895 35019458

[41] Chen Q Xu L Liang C Wang C Peng R Liu Z. Photothermal Therapy With Immune-Adjuvant Nanoparticles Together With Checkpoint Blockade for Effective Cancer Immunotherapy. Nat Commun (2016) 7:13193. doi: 10.1038/ncomms13193 27767031 PMC5078754

