# Supplementary material for: An Injectable Hydrogel for Enhanced FeGA-Based Chemodynamic Therapy by Increasing Intracellular Acidity
Source: Front Oncol. 2021 Sep 22;11:750855. doi: 10.3389/fonc.2021.750855 (PMC8492932; doi:10.3389/fonc.2021.750855)
Supplement: Supplementary Figures S1-4 — Representative immunofluorescence images of ROS in tumor slices. [file DataSheet_1.docx]

Experimental Procedures

**Materials and reagents.**

Iron chloride hexahydrate (FeCl_3_·6H2O) and polyvinylpyrrolidone K30 (PVP) were purchased from Sinopharm Chemical Reagent Co., Ltd. Agarose was purchased from Yare Shanghai. Gallic acid and α-cyano-4-hydroxycinnamic acid (α-CHCA) were acquired from Macklin. Reactive Oxygen Species Assay Kit, and MTT Cell Proliferation Assay Kit were obtained from Yeasen Biotech Co., Ltd (China). Lactic Acid Assay Kit was provided by Nanjing Jiancheng Bioengineering Institute (China). The other reagents used in this work were purchased from Sinopharm Chemical Reagent (China) and Aladdin-Reagent (China).

**Cell culture**

4T1 mouse breast cancer cell line was obtained from the Cell Bank of the Chinese Academy of Sciences and incubated in RPMI-1640 medium supplemented with 10% FBS in a humidified atmosphere at 37℃.

**Preparation and characterization of Fe-GA nanoparticles (FeGA)**

Fresh GA–Fe was prepared according to the literature.([1](#_ENREF_1)) FeCl3·6H2O was added into the gallate solution to keep a 1:1 stoichiometry of Fe^3+^ and GA^4−^ for reaction with magnetic stirring and nitrogen supplementation for 1 h, after which the suspension was centrifuged and the precipitate was washed, collected, and stored for further use. The morphology structures of FeGA were observed by the TEM (JEOL-2100). UV-vis spectra of different samples were recorded by the UV-vis spectrophotometry Lambda 35 (Perkin-Elmer). XPS spectra were recorded by ESCAlab250 (Thermal Scientific). Zeta potential of FeGA were measured by dynamic light scattering.

**Preparation and characterization of acid-enhanced CDT system (AES)**

The general protocol for the hydrogel preparation is as follows. The prepared FeGA (10 mg/mL in PBS) and the α-CHCA (10 mg/mL in DMSO) were mixed into 2% agarose solution to form AES. Wherein the concentration of FeGA and α-CHCA was 200 and 20 μg/mL, respectively, and the DMSO content in AES was no more than 0.5 %. Scanning electron microscopy (SEM) images were captured on a Hitachi FE-SEM S4800 instrument with an acceleration voltage of 3 kV.

**Rheological Test**

Rheology experiments were performed on an Anton Paar rheometer. Hydrogel samples of different temperatures were prepared and gently placed on the middle of a 15 mm diameter parallel plate with a proper gap. Dynamic oscillatory frequency sweep measurements were conducted at a 1% strain amplitude. To prevent the evaporation of water, a lid was prepared on the top.

**Photothermal conversion ability of FeGA.**

Different concentration of FeGA PBS solution (0, 25, 50, 100 and 200 μg/mL) were irradiated by 808 nm laser (0.5 W/cm^2^) for 5min. Through the infrared thermal imaging camera, the photothermal image of the suspension is performed at 30 s intervals.

**α-CHCA release study**

The *in vitro* α-CHCA release profile from AES was carried out. 1mL of AES containing 20 μg α-CHCA was added into culture dish. To investigate the stimuli effect of laser irradiation on the release behavior, the release experiment of α-CHCA was initially performed with or without 0.5 W/cm^2^ 808 nm laser irradiation for 5 min. At appropriate time point, 100 μL of different samples were collected, and an UV−vis spectrophotometer was used to monitor the released α-CHCA content.

**Intracellular reactive oxygen species (ROS) generation**

For determination of ROS levels via fluorescent imaging, 4T1 cells were incubated with 5 different groups: (1) PBS; (2) Near infrared laser irradiation (808nm laser, 0.5W/cm^2^); (3) AES; (4) AES+NIR and (5) FeGA+NIR. The α-CHCA concentration was 20 μg/mL in group 3, and 4. The FeGA concentration was 200 μg/mL in group 3 and 5. In order to facilitate the study of ROS production, all samples were added directly into the cells. Then, the fluorescent dye, DCFH-DA (10 μmol/L), was added and co-incubated for 20 min at 37 °C. Then, cells in group 2, 4 and 5 were irradiated with the NIR. ROS level was determined by a confocal laser scanning microscope (CLSM; IX81, Olympus, Japan).

**Intracellular lactate accumulation**.

4T1 cells were seeded into the 48-well plate (2 × 10^4^ cells per well). After cultured for 12 h, the cell supernatant was displaced with the fresh culture medium and treated with 5 different groups: (1) PBS; (2) Near infrared laser irradiation (808nm laser, 0.5W/cm^2^); (3) AES; (4) AES+NIR and (5) FeGA+NIR. The α-CHCA concentration was 20 μg/mL in group 3, and 4. The FeGA concentration was 200 μg/mL in group 3 and 5. After 4 h, cells with different treatments were collected, and lactate content was detected using the Lactic Acid assay Kit.

**Photothermal Conversion Efficiency**

An 808 nm NIR laser (Changchun New Industries Tech.Co., Ltd., Changchun, China)

with irradiation powers was used to stimulate the concentrations of FeGA (200 ug/mL) in an aqueous medium. The photothermal images of the FeGA-based suspensions during laser irradiation were recorded every 30 s using an infrared thermal imaging system. The NIR laser source was equipped with a 4 mm diameter laser module with an adjustable power. The photothermal conversion efficiency was calculated using the following equation:


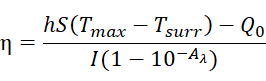


where h is the heat transfer coefficient, S is the surface of the container, T_max_ and T_surr_ are the equilibrium temperature and ambient temperature, respectively. Q_0_ is the heat associated with the light absorbance of the solvent, A_λ_ is the absorbance of FeGA at 808 nm, and I is the laser power density. According to the above equation, the η value of FeGA was determined to be about 42.6%.

***In vitro* phototoxicity of AES**

The phototoxicity was measured by MTT assay. 4T1 cells were seeded in 96-well plates at a density of 5 × 10^3^ cells per well and incubated for 24 h. Afterwards, 4T1 cells were incubated for 6 different groups: 4T1 cells were incubated with 5 different groups: (1) PBS; (2) Near infrared laser irradiation (808nm laser, 0.5W/cm^2^); (3) AES; (4) AES+NIR and (5) FeGA+NIR. The α-CHCA concentration was 20 μg/mL in group 3, and 4. The FeGA concentration was 200 μg/mL in group 3 and 5. Then, cells in group 2, 4 and 5 were irradiated with the NIR. At the end of the incubation, 5 mg/mL MTT solution was added, and the plate was incubated for another 4 h. Finally, the absorbance values of the cells were determined by using a microplate reader (Emax Precision, USA) at 570 nm. The background absorbance of the well plate was measured and subtracted. The cytotoxicity was calculated by dividing the optical density (OD) values of treated groups (T) by the OD values of the control (C) (T/C × 100%). ([2](#_ENREF_2), [3](#_ENREF_3))

**Biocompatibility of FeGA**

We then tested the biocompatibility of FeGA and α-CHCA to other types of cells under dark condition. 4T1 cancer cells were seeded in 96-well plates at a density of 5 × 10^3^ cells per well and incubated for 24 h. Afterwards, cells were incubated for 6h with different concentrations of FeGA (0, 50, 100 and 200 μg/mL). At the end of the incubation, 5 mg/mL MTT solution was added, and the plate was incubated for another 4 h. Finally, the absorbance values of the cells were determined by using a microplate reader (Emax Precision, USA) at 570 nm. The background absorbance of the well plate was measured and subtracted. The cytotoxicity was calculated by dividing the optical density (OD) values of treated groups (T) by the OD values of the control (C) (T/C × 100%).

**Animal tumor models**

Female BALB/c nude mice aged 4-5 week were purchased from Vital River Company (Beijing, China). 100 μL of 4T1 cell suspension (1×10^6^ cells per mL) were subcutaneous injected into each mouse to establish the tumor models. The animal experiments were carried out according to the protocol approved by the Ministry of Health in People’s Republic of PR China and were approved by the Administrative Committee on Animal Research of the second clinical Medicine College of Jinan University.

***In vivo* infrared thermography**

To monitor the *in vivo* photothermal effect, AES (FeGA: 1 mg/kg, α-CHCA: 0.1 mg/kg) was intratumorally injected into the tumor-bearing mice, and then the tumors suffered from 0.5 W/cm^2^ irradiation for 5min at 1 h post-injection. PBS injection used as control group. Meanwhile, the temperature at the tumor was monitored using an infrared camera (Fotric 225).

***In vivo* antitumor study**

The mice were firstly divided randomly into 6 groups (each group included 5 mice): 1) a control group (PBS injection); 2) AES; 3) NIR; 4) AES+NIR. The injection method is intratumoral injection. Among them, the dose of FeGA and α-CHCA in groups 2 and 4 are 1 mg/kg and 0.1 mg/kg, respectively. NIR was conducted 1h after the injection. Mice body weight and tumor volume in all groups were monitored every 5 days. A caliper was employed to measure the tumor length and tumor width and the tumor volume was calculated according to following formula. Tumor volume = tumor length × tumor width^2^ / 2. After 15 days treatment, mice were sacrificed. Five main organs (heart, liver, spleen, lung and kidney) of all mice were harvested, washed with PBS, and fixed with paraformaldehyde for histology analysis. The blood samples from these mice (≈1 mL) were collected for blood biochemistry analysis. And the tumor tissues were weighed, and fixed in 4% neutral buffered formalin, processed routinely into paraffin, and sectioned at 4 μm. Then the sections were stained with hematoxylin and eosin (H&E) and TUNEL and finally examined by using an optical microscope (BX51, Olympus, Japan).

**Statistical analysis**

Data analyses were conducted using the GraphPad Prism 5.0 software. Significance between every two groups was calculated by the Student’s t-test. *P < 0.05, **P < 0.01, ***P < 0.005.

**References**

1. B. Yang, H. Yao, H. Tian, Z. Yu, Y. Guo, Y. Wang, J. Yang, C. Chen and J. Shi: Intratumoral synthesis of nano-metalchelate for tumor catalytic therapy by ligand field-enhanced coordination. *Nat. Commun.*, 12(1), 3393 (2021) doi:10.1038/s41467-021-23710-y

2. D. Zhu, M. Lyu, W. Jiang, M. Suo, Q. Huang and K. Li: A biomimetic nanozyme/camptothecin hybrid system for synergistically enhanced radiotherapy. *J Mater Chem B*, 8, 5312-5319 (2020) doi:10.1039/d0tb00676a

3. D. Zhu, M. Lyu, Q. Huang, M. Suo, Y. Liu, W. Jiang, Y. Duo and K. Fan: Stellate Plasmonic Exosomes for Penetrative Targeting Tumor NIR-II Thermo-Radiotherapy. *ACS Appl. Mater. Interfaces*, 12(33), 36928-36937 (2020) doi:10.1021/acsami.0c09969


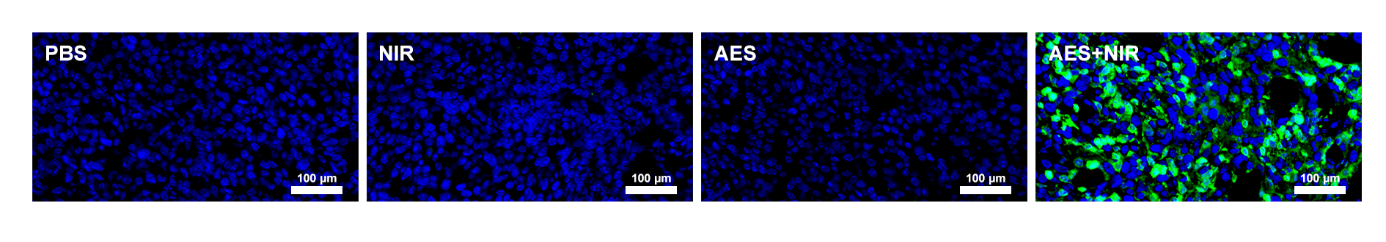


**Figure S1**. Representative immunofluorescence images of ROS in tumor slices.
